# Oral PrEP continuation rates among key populations in Nigeria: a retrospective cohort study of a large-scale HIV prevention programme

**DOI:** 10.1136/bmjgh-2024-018739

**Published:** 2025-12-19

**Authors:** Kene David Nwosu, Abiye Kalaiwo, Wingston Ng’ambi, Omosalewa Oyelaran, Paul Umoh, Janne Estill, Olivia Keiser

**Affiliations:** 1 Institute of Global Health, University of Geneva, Geneva, Switzerland; 2 Office of HIV/AIDS and TB, US Agency for International Development, Abuja, Nigeria; 3 Department of Health Systems, Kamuzu University of Health Sciences, Blantyre, Malawi; 4 Heartland Alliance Nigeria, Abuja, Nigeria

**Keywords:** HIV, Chemoprophylaxis, Epidemiology

## Abstract

**Introduction:**

Pre-exposure prophylaxis (PrEP) is an effective HIV prevention strategy, but its impact is limited by low uptake and poor continuation rates. This study investigates oral PrEP continuation among key populations (KPs) enrolled in an HIV prevention programme across seven states in Nigeria, representing one of the largest longitudinal PrEP continuation studies in Sub-Saharan Africa to date.

**Methods:**

We analysed data from 43 788 clients who initiated daily oral PrEP between January 2020 and March 2023. The sample comprised female sex workers (20 574, 47.0%), men who have sex with men (12 946, 29.6%), people who inject drugs (9462, 21.6%) and transgender individuals (806, 1.8%). The primary outcome was 6-month PrEP continuation, defined as having a recorded PrEP refill more than 6 months after initiation. We used multivariable mixed-effects logistic regression to identify factors associated with PrEP continuation; the model included KP group, age, education, occupation, marital status and time since PrEP initiation and accounted for clustering at the facility level.

**Results:**

Among the 43 788 clients initiating PrEP, the 6-month continuation rate was 11.5%. Female sex workers had the highest 6-month continuation rate (13.7%), while transgender individuals had the lowest (3.5%; adjusted OR (aOR) 0.37 (0.24–0.55), compared with female sex workers). The continuation rate generally increased with age; the oldest clients (≥40 years) had a significantly higher continuation rate than those aged 18–24 (12.9% vs 11.0%; aOR 1.15 (1.02–1.29)). Unemployment was also associated with a small but significant reduction in continuation, with a rate of 11.8% for unemployed clients compared with 12.1% for employed clients (aOR 0.86 (0.79–0.94)).

**Conclusion:**

These findings highlight the need for targeted interventions to improve PrEP continuation among KPs in Nigeria, particularly for younger clients and transgender people.

**WHAT IS ALREADY KNOWN ON THIS TOPIC**

- Pre-exposure prophylaxis (PrEP) is an effective HIV prevention strategy, with consistent use greatly reducing the risk of HIV acquisition.

**WHAT THIS STUDY ADDS**

- Despite a provider-facilitated approach, this study reveals substantial attrition from PrEP programmes among key populations in Nigeria within the first 6 months.

**HOW THIS STUDY MIGHT AFFECT RESEARCH, PRACTICE OR POLICY**

- These findings suggest a need for strengthening PrEP programme design and implementation, incorporating strategies that promote PrEP continuation through ongoing support and tailored services.

## Introduction

 HIV pre-exposure prophylaxis (PrEP) involves antiretroviral medications used by HIV-negative individuals to reduce HIV acquisition risk. The most common PrEP regimens consist of daily oral pills of tenofovir disoproxil fumarate combined with either emtricitabine (FTC) or lamivudine (3TC).^1^ Randomised controlled trials show these regimens can reduce HIV infection risk by up to 92% when taken consistently.^2^,^4^

Despite high PrEP efficacy, there remain challenges with implementation, particularly in access and adherence. In 2020, only 24.7% of the estimated 1.2 million people with PrEP indications in the USA were prescribed it,^5^ and coverage is even lower in low and middle-income countries.^1^ The limited uptake of PrEP is compounded by poor continuation rates among those who do initiate treatment. A pooled systematic review found that 70% of PrEP users across studies had discontinued treatment or shown suboptimal adherence within 6 months of initiation,^6^ with factors like side effects, social stigma and logistical challenges cited as reasons for discontinuation.^7^ These barriers underscore the need for comprehensive strategies to expand PrEP availability and support long-term PrEP continuation.

Nigeria has the fourth largest HIV epidemic globally, with a particularly severe epidemic among key populations (KPs).^8 9^ These groups, which include female sex workers (FSWs), men who have sex with men (MSM), transgender individuals and people who inject drugs (PWIDs), have significantly higher HIV prevalence compared with the general population.^10^ For example, a 2020 survey of several states in Nigeria found an HIV prevalence of 16% among FSWs and 25% among MSM,^10^ compared with 1.3% among the general adult population. The disproportionate burden underscores the importance of targeted HIV prevention efforts for KPs.

Given the burden of HIV among KPs in Nigeria, PrEP rollout for these groups is a critical component of the country’s HIV prevention strategy,^11^ and several implementing partners have active oral PrEP programmes for KPs. However, data on PrEP continuation rates and its determinants in these contexts are limited. This lack of information hinders the development of evidence-based interventions to improve PrEP access and continuation among KPs.

The current study addresses this gap by investigating PrEP continuation rates among KPs enrolled in an HIV prevention programme across seven Nigerian states. By analysing data from a large sample of PrEP users, the study provides insights into the patterns of PrEP continuation in the Nigerian context. Our findings will inform the development of targeted interventions for HIV prevention among KPs in Nigeria and similar settings.

## Materials and methods

### Study setting and population

This study analysed routine programme data from the Key Population Community HIV Services for Action and Response (KP CARE 1) PrEP programme in Nigeria implemented by Heartland Alliance LTD/GTE. The KP CARE 1 PrEP programme operates across seven Nigerian states: Akwa Ibom, Bayelsa, Cross River, Jigawa, Lagos, Niger and Yobe. It employs a hotspot and venue-based outreach approach to engage FSWs, MSM, transgender individuals, PWIDs, those in incarceration and partners of individuals that identify as a member of a KP.

The KP CARE 1 programme uses strategically located ‘One Stop Shops’ (OSS) to provide comprehensive HIV services. These centres combine a central facility with mobile outreach services to enhance accessibility. Each OSS is staffed by a team of healthcare providers, including medical professionals and support staff, to offer a range of services from HIV testing and counselling to PrEP and Antiretroviral Therapy (ART) initiation and follow-up.

HIV testing at these facilities followed Nigeria’s national HIV testing services (HTS) algorithm, which employs a serial rapid diagnostic strategy consisting of two sequential antibody-based rapid tests: an initial screening assay followed by a confirmatory assay in cases where the first test result is reactive.^12^ If individuals test HIV-negative, they are offered oral PrEP as part of an HIV prevention package. For those willing to initiate PrEP, eligibility is confirmed through assessment of renal function (creatinine clearance above 60 mL/min) and absence of known allergies or contraindications to the PrEP components.

Eligible clients are initially dispensed a 1 month supply of PrEP, with a follow-up scheduled around 4 weeks later to review adherence and possible side effects. If stable, clients are ideally transitioned to a routine 3 monthly refill schedule, in line with Nigeria’s national HIV prevention guidelines.^13^ This ideal schedule can be adjusted based on client mobility, local logistics or service delivery modality, particularly in mobile or outreach settings.

Clients are scheduled for regular follow-up visits at the OSS for clinical monitoring and PrEP medication refills. These visits are designed to ensure continuity of care, assess adherence and address any concerns or side effects. PrEP-related clinical encounters are documented in the electronic register.

For this analysis, we included data from clients who initiated oral PrEP between 1 January 2020 and 15 March 2023 and who were at least 18 years old at the time of initiation. The data were exported from the electronic programme register on 15 March 2024, allowing for a minimum of 12 months of follow-up.

### Statistical analysis

The primary outcome of this study was 6-month PrEP continuation, defined as having a documented PrEP refill visit at any point beyond 6 months (≥183 days) from the date of PrEP initiation. This timepoint was chosen based on guidance from other PrEP continuation studies and represents a clinically meaningful duration for assessing sustained engagement with PrEP services.

Covariates were selected based on availability in the electronic programme register and relevance to PrEP continuation. These included KP group, age group, highest education level, occupation and marital status. KP groups are classified as FSWs, PWIDs, MSM and transgender individuals based on primary self-identified group as recorded during initial programme enrolment. Age was categorised into five groups: 18–24, 25–29, 30–34, 35–39 and 40+ years. Highest education level was classified as secondary or less, or post-secondary. Occupation was categorised as employed, not employed or student. Marital status was dichotomised as never married or ever married.

To enhance data quality, we performed several checks, including the deduplication of client identifiers and logic checks for date and categorical variables. Any identified inconsistencies were flagged and resolved in collaboration with the programme’s monitoring and evaluation officers.

To address missing data in education, occupation and marital status, we employed multiple imputation by chained equations (MICE) using predictive mean matching with 10 imputations. The imputation model included all complete covariates (KP group, age group, sex, time since PrEP initiation and facility) as well as the outcome variable (6-month PrEP continuation) as predictors. This imputed dataset was used for both univariable and multivariable analyses.

For univariable analyses, we applied mixed-effects logistic regression models with a random intercept for facility to account for clustering. Each covariate was analysed separately against the outcome of 6-month PrEP continuation. These models were run on each of the 10 imputed datasets, and results were pooled using Rubin’s rules.

The multivariable analysis employed a mixed-effects logistic regression model with a random intercept for facility. Only covariates with a global χ² test p value <0.30 in the univariable analysis were retained for the multivariable model and for visualisation. Because programme performance and service-delivery strategies may have evolved over the study period, time since PrEP initiation was also included as a control variable in the multivariable model. This was calculated as the number of days between the date of first PrEP pickup and the date of data extraction, and categorised into 12–16 months, 16–20 months, 20–24 months, 24–28 months and 28+ months. The multivariable model was also applied to each imputed dataset, with results pooled across imputations.

We calculated and reported both unadjusted (from univariable analyses) and adjusted (from multivariable analysis) ORs with 95% CIs to quantify associations between covariates and PrEP continuation.

All analyses and visualisations were performed using R statistical software (V.4.2.2), with the ‘mice’ package for multiple imputation and the ‘lme4’ package for mixed-effects modelling.

### Ethical considerations

This study used de-identified, routinely collected data from the KP CARE 1 PrEP programme in Nigeria, implemented by Heartland Alliance LTD/GTE. Ethical approval was obtained from the Federal Capital Territory Health Research Ethics Committee, Nigeria (approval no: FHREC/2023/01/127/20-07-23). In accordance with Nigerian HTS policy, written informed consent was obtained from all clients for both service provision and potential use of de-identified data for research purposes.

### Patient and public involvement

Patients or the public were not involved in the design or reporting of this research.

### Funding

This study was supported by the Swiss National Science Foundation (grants nos. 163 878 and 320030_192452) and the United States Agency for International Development. The funders of the study had no role in study design, data collection, data analysis, data interpretation or writing of the report.

## Results

### Sample characteristics

**Figure 1:**
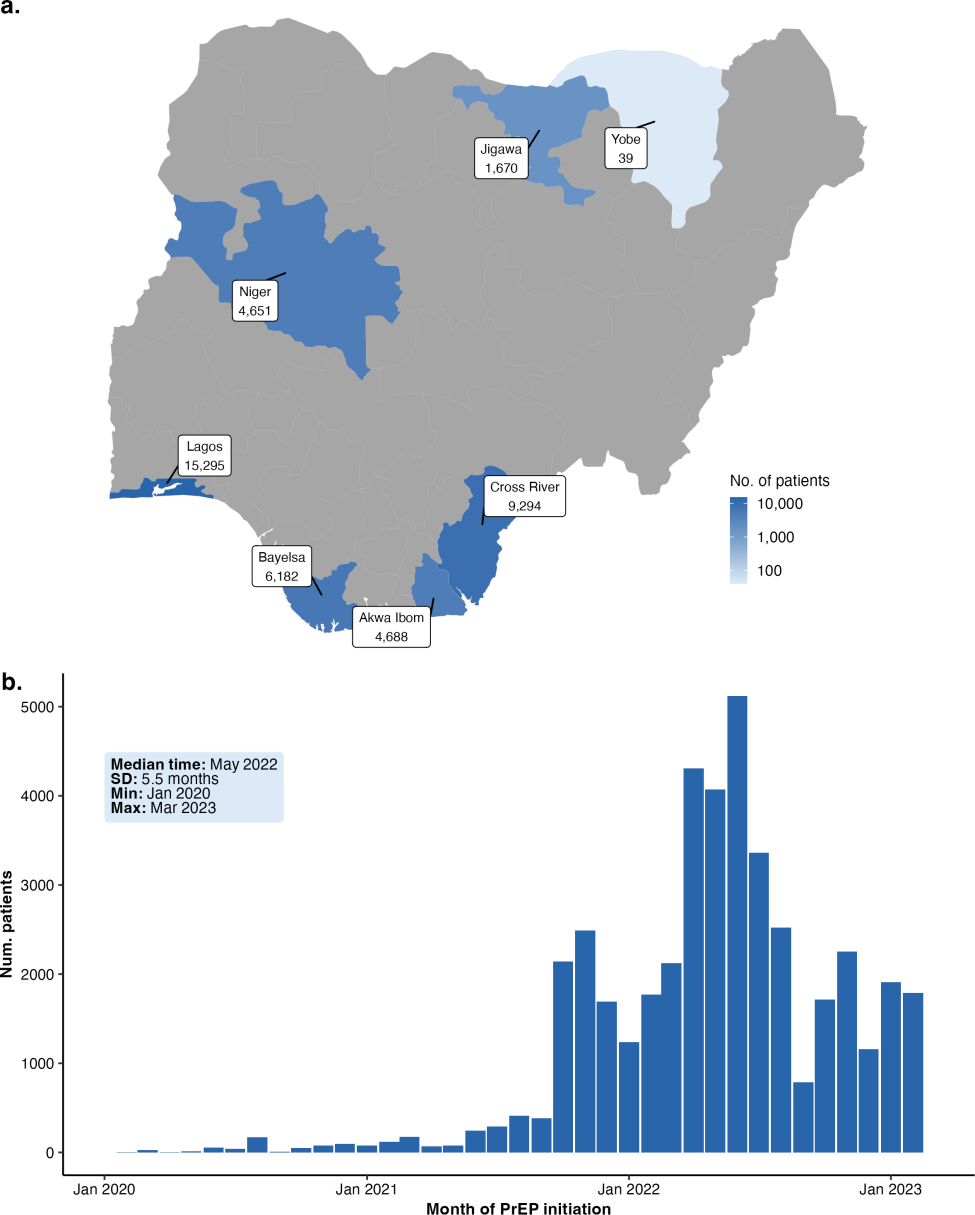
Descriptive characteristics of PrEP initiation among key populations (KPs) in Nigeria. (**a**) Geographic distribution of PrEP clients across Nigerian states. Colour intensity indicates the number of clients. (**b**) Timeline of PrEP initiation from January 2020 to the latest available date, showing the number of clients starting PrEP each month. PrEP, pre-exposure prophylaxis.

The analysis included 43 788 clients who initiated PrEP between 1 January 2020 and 15 March 2023, across seven states in Nigeria (table 1; figure 1a; Online supplemental figure 1). The sample comprised 53.4% females (n=23 387) and 46.6% males (n=20 401).

**Table 1:** Characteristics of the study sample

Characteristic	N	Percent
Total	43 788	–
Sex		NA
Female	23 387	53.4
Male	20 401	46.6
Key population		NA
Female sex workers	20 574	47.0
People who inject drugs	9462	21.6
Men who have sex with men	12 946	29.6
Transgender individuals	806	1.8
Age group (years)		NA
18–24	17 367	39.7
25–29	12 557	28.7
30–34	6115	14.0
35–39	4165	9.5
40+	3584	8.2
Year of PrEP initiation		NA
2020	547	1.2
2021	8167	18.7
2022	30 423	69.5
2023	4651	10.6
State of residence		NA
Akwa Ibom	4688	10.7
Bayelsa	6182	14.1
Cross River	9294	21.2
Jigawa	1670	3.8
Lagos	15 295	34.9
Niger	4651	10.6
Yobe	39	0.1
Missing state of residence	1969	4.5
Marital status		NA
Never married	41 819	95.5
Ever married	1840	4.2
Missing marital status	129	0.3
Education		NA
Secondary or less	32 004	73.1
Post-secondary	6491	14.8
Missing education	5293	12.1
Employment status		NA
Employed	21 657	49.5
Not employed	13 386	30.6
Student	6984	15.9
Missing employment status	1761	4.0

N is the number of individuals in each stratum.

PrEP, pre-exposure prophylaxis.

The largest KP group was FSWs (47.0%, n=20 574), followed by MSM (29.6%, n=12 946) and PWIDs (21.6%, n=9462). A small proportion were transgender individuals (1.8%, n=806).

Most clients were young adults, with over half (68.4%, n=29 924) aged between 18 and 29 years. The majority had secondary education or less as their highest level of education (73.1%, n=32 004), and about half reported being employed (49.5%, n=21 657). Almost all clients had never been married (95.5%, n=41 819).

The largest proportion of clients resided in Lagos state (34.9%, n=15 295), followed by Cross River (21.2%, n=9294) and Bayelsa (14.1%, n=6182). Most clients initiated PrEP in 2022 (69.5%, n=30 423), reflecting an expansion of the programme during that period (figure 1b; Online supplemental figure 2).

### PrEP continuation

**Figure 2:**
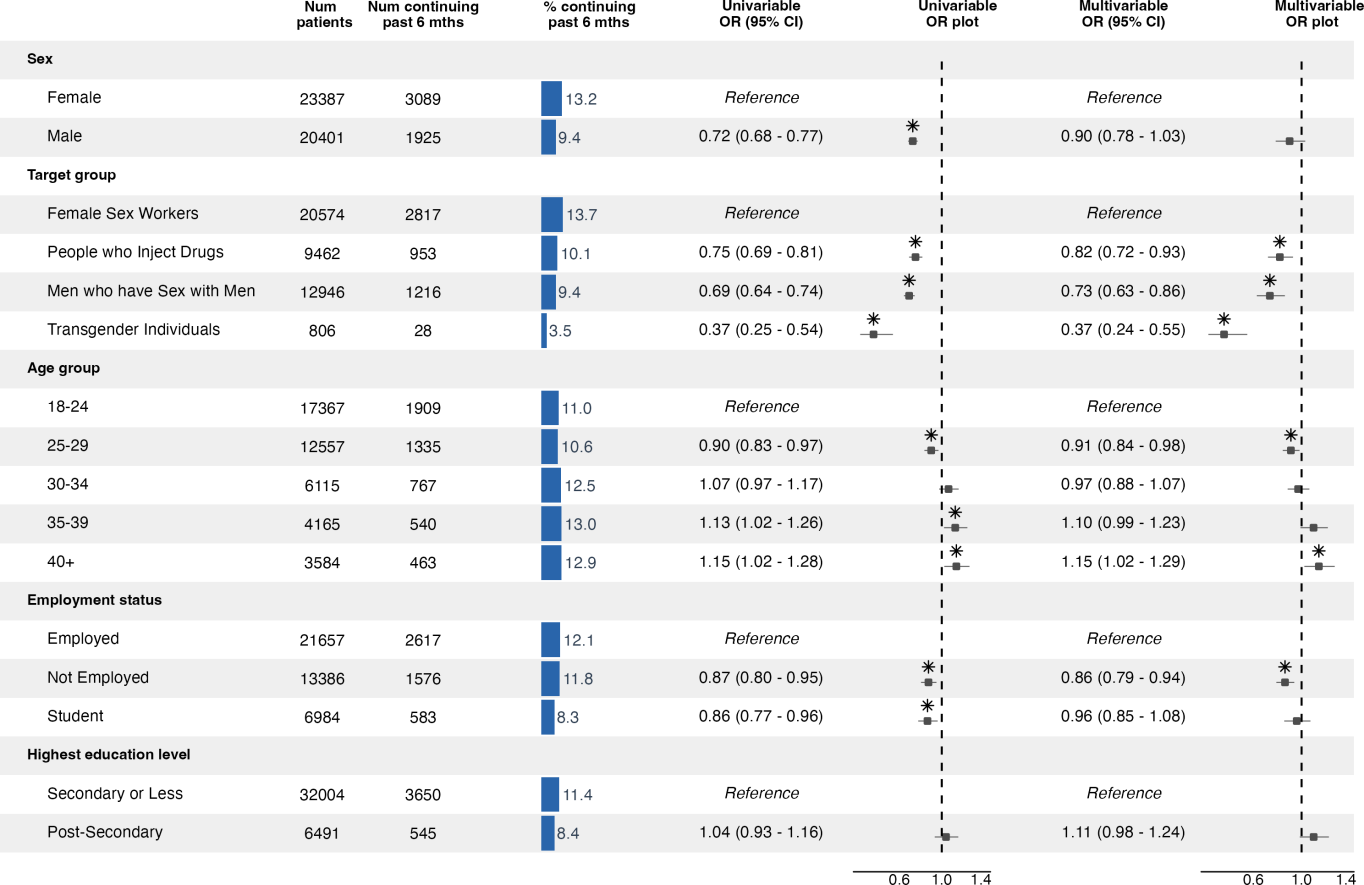
Factors associated with PrEP continuation past 6 months among key populations in Nigeria. Based on multivariable mixed-effects logistic regression analysis with random intercepts for the service delivery site. ORs and 95% CIs represent pooled estimates from 10 multiply imputed datasets using Rubin’s rules. Box-whisker plots indicate ORs and 95% CIs. Asterisks indicate statistical significance at a 0.05 alpha level. PrEP, pre-exposure prophylaxis.

Among the 43 788 clients who initiated PrEP, the overall 6-month PrEP continuation rate was 11.5% (5014 of 43 788). Multivariable analysis identified KP group, age and employment status as significantly associated with 6-month PrEP continuation (figure 2; Online supplemental figure 3).

Sex-based analysis revealed that male clients had a slightly lower 6-month continuation rate (9.4%) compared with female clients (13.2). However, when adjusting for other factors, the association between continuation rate and sex was not statistically significant (male adjusted OR (aOR) 0.90 (0.78–1.03)).

KP group was the strongest predictor of 6-month PrEP continuation. FSWs had the highest rate (13.7%). Compared with FSWs, all other groups had significantly lower rates of continuing PrEP. PWIDs had a continuation rate of 10.1% (aOR 0.82 (0.72–0.93)), while MSM had a rate of 9.4% (aOR 0.73 (0.63–0.86)). Transgender individuals had the lowest continuation rate, substantially below other groups, at just 3.5% (aOR 0.37 (0.24–0.55)).

Age was also a significant factor, with continuation rates generally increasing with age. The youngest group (18–24 years) had a continuation rate of 11.0%, while clients aged 25–29 had a 10.6% continuation rate (aOR 0.91 (0.84–0.98)). Older clients had higher continuation rates: those aged 35–39 had 13.0% (aOR 1.10 (0.99–1.23)), and those aged 40+ had 12.9% (aOR 1.15 (1.02–1.29)).

Employment status was significantly associated with 6-month PrEP continuation. Unemployed clients had significantly lower PrEP continuation rates compared with employed clients (11.8% vs 12.1%; aOR 0.86 (0.79–0.94)).

Education level was not significantly associated with 6-month PrEP continuation. Clients with post-secondary education had an 8.4% continuation rate compared with 11.4% for those with secondary education or less, a difference that was not statistically significant (aOR 1.11 (0.98–1.24)).

## Discussion

Our study investigated oral PrEP continuation among KPs enrolled in an HIV prevention programme in seven states in Nigeria. We observed a low overall continuation rate, with 11.5% maintaining PrEP use beyond 6 months post-initiation. Other studies in similar settings have also reported substantial drop-offs in PrEP continuation. An analysis of routine programme data of MSM and FSWs on oral PrEP in Cameroon found a continuation rate of 28% at 6 months.^14^ Similarly, a study on persons at high HIV risk in Zambia reported continuation rates of 25% and 11% at 6 and 12 months, respectively.^15^

However, the 6-month continuation rate reported in our study, at 11.5%, is still substantially lower than these comparable studies. One potential explanation is the provider-facilitated approach employed in the Nigerian KP PrEP programme, where individuals are actively encouraged to start PrEP immediately following a negative HIV test result. While this approach effectively promotes initial uptake of PrEP among at-risk individuals, the low continuation rates indicate significant challenges in translating initial uptake into sustained use.

It is important to interpret these low PrEP continuation rates cautiously. While concerning, they do not necessarily equate to programme failure or lack of individual engagement with HIV prevention. KPs often experience fluctuating HIV risk, which may necessitate intermittent rather than continuous PrEP use.^16^ Clients may also permanently reduce their risk behaviours, eliminating the need for ongoing PrEP.^17^ Future research should therefore aim to distinguish between discontinuation due to poor adherence and discontinuation due to intentional, risk-based cycling off PrEP or changes in risk behaviour. This distinction will be crucial in guiding programme development and evaluation.

In our study, PrEP continuation rates varied significantly across different KP groups, with transgender individuals displaying extremely low 6-month continuation rates at 3.5%. This disparity may stem from higher levels of stigma, mistrust of healthcare providers and competing health priorities unique to the transgender community.^18^ Addressing these challenges is crucial for improving PrEP continuation among transgender people.

Age was positively associated with 6-month continuation, with older age groups having higher odds compared with younger groups. This finding aligns with results from other studies^19 20^ and may suggest greater health literacy or self-efficacy among older individuals. For younger populations, leveraging digital health solutions, such as mobile apps for medication counselling, reminders and consultations, could be effective to improve continuation rates.^21^

We also observed that unemployment was associated with a statistically significant reduction in 6-month PrEP continuation (aOR 0.86 (0.79–0.94)). Similar patterns have been reported in other settings. In a study in Namibia, unemployed clients had 73% lower odds of remaining in PrEP care at 3 months,^22^ and a US cohort study among MSM in New York City found that employed individuals had 69% higher odds of sex-related PrEP adherence.^23^ A recent meta-ethnographic scoping review of young MSM summarised financial burdens—such as transport costs, pharmacy fees and lost wages—as barriers to both adherence and retention.^24^ Taken together, these findings suggest that economic insecurity may disrupt the routines and resources required for sustained PrEP use. Future programme adaptations might explore how flexible refill options or integrated economic support can help mitigate these challenges.

Our study has several limitations that affect the interpretation of its findings. First, our definition of continuation, based on pharmacy refill data, is an imperfect proxy for PrEP use. Future studies could incorporate more robust adherence measures (eg, self-report, pill counts, drug level monitoring) alongside refill data. Second, we lacked data on individual-level risk behaviours and reasons for discontinuation, which could provide valuable context for interpreting PrEP continuation patterns. Qualitative investigations into reasons for discontinuation would be beneficial in future studies. Finally, our study relied on routinely collected programme data, which may be prone to measurement error. We sought to mitigate this by performing data quality checks, such as deduplicating records and conducting logic checks, and by using multiple imputation to handle missing data. While these measures strengthen our analysis, some residual data quality issues could still affect the precision of our findings.

Despite these limitations, this study has notable strengths. The large sample size of 43 788 clients, one of the largest in studies of this kind, provides robust estimates of PrEP continuation rates across diverse KPs. Furthermore, the use of real-world programme data offers valuable insights into PrEP use patterns under actual implementation conditions, enhancing the external validity of our findings. Notably, our study was able to examine PrEP continuation rates among transgender individuals—a group often underrepresented in research due to small sample sizes—highlighting significant disparities and the need for targeted interventions.

## Conclusions

Our study reveals key insights into oral PrEP use patterns among KPs in Nigeria. While the provider-facilitated approach used in this KP programme effectively promotes initial PrEP uptake, the low continuation rates signal a need for interventions to support sustained adherence. Specific strategies tailored to vulnerable groups like transgender individuals, who face unique challenges, are essential.

Ultimately, improving PrEP continuation rates is crucial for realising the full potential of this HIV prevention tool. Concerted efforts are needed to address the barriers to sustained use and ensure that KPs can effectively leverage PrEP to reduce their HIV risk over the long term.

## Supplementary material

10.1136/bmjgh-2024-018739 online supplemental figure 1

10.1136/bmjgh-2024-018739 online supplemental figure 2

10.1136/bmjgh-2024-018739 online supplemental figure 3

## Data Availability

Summarized data relevant to the study are included in the article or uploaded as supplementary information. Anonymised individualised line list data can be made available upon request.
